# Exploring physicians’ emotional reactions to suicidal patients: the impact of physician- and patient-related issues

**DOI:** 10.1186/s12991-025-00602-9

**Published:** 2025-10-24

**Authors:** Dimitrios Kiakos, Céline Bourquin-Sachse, Stéphane Richard-Devantoy, Friedrich Stiefel, Laurent Michaud

**Affiliations:** 1 General Psychiatry Service, Lausanne University Hospital (CHUV), 18, Place Chauderon, Lausanne, 1003 Switzerland; 2 Liaison Psychiatry Service, Lausanne University Hospital (CHUV), Lausanne, Switzerland; 3 McGill Group for Suicide Studies, Department of Psychiatry, McGill University, Montreal, Canada; 4 CISSS Des Laurentides, St-Jerome, Canada

**Keywords:** Suicide, Suicide risk, Physician–patient relationship, Countertransference, Therapeutic alliance

## Abstract

**Background:**

The physician–patient relationship is essential in the care of suicidal patients, yet factors shaping this relationship remain insufficiently explored. This study aimed to explore physicians’ emotional reactions to suicidal patients and how both physician- and patient-related issues influence these responses.

**Methods:**

Interviews were conducted with six physicians from the Douglas Mental Health University Institute in Montreal. A thematic analysis was performed using Hayes’ structural model of countertransference as the analytical framework.

**Results:**

Three primary emotional reactions emerged: emotional connection/avoidance, confidence/doubts, and powerlessness attributed to own limitations/to the patient. Clinicians’ core needs—the need to help, need for security, and need for efficacy—were found to be pivotal in shaping these emotional responses. Similarly, patient-related factors, notably life experiences, disease, suicidality, and attitudes significantly influenced these reactions. Patterns linking physicians’ emotional responses to their underlying needs and patient-related factors were analyzed, leading to the development of a conceptual framework.

**Conclusions:**

This framework offers implications for research, clinical supervision, and medical training, fostering deeper insight into the physician–patient relationship in the context of suicidality.

**Supplementary Information:**

The online version contains supplementary material available at 10.1186/s12991-025-00602-9.

## Background

Suicide is a major public health concern, with over 700,000 people taking their own lives every year and many others attempting suicide. In 2021, suicide ranked as the third leading cause of death among individuals aged less than 30 worldwide. Moreover, each suicide leaves a profound and lasting impact on those left behind. [44].

The biomedical model [25], widely used as the reference framework for assessing and preventing suicide risk, has been linked to low levels of patient engagement in treatment [12, 22, 23]. Therefore, patient-centered collaborative models (CAMS, ASSIP, Finn’s) [5, 11, 20] recognizing the importance of the therapeutic relationship and actively engaging patients in their own care, have been developed. Nevertheless, establishing a therapeutic alliance with suicidal patients remains a challenging endeavor for clinicians. Studies [2, 32, 35] reported negative attitudes of healthcare professionals towards suicidal patients, influencing the way patients perceived care [30, 40].

The psychoanalytic perspective of transference and countertransference provided a useful framework to understand the challenges and emotional reactions of clinicians encountering suicidal patients. [7] as an unconscious reaction of the analyst to the patient’s transference having detrimental effects on therapy, the concept of countertransference has expanded over time to encompass all therapist’s or clinician’s reactions to a patient [8], and beyond the psychotherapeutic setting. Theoretical reflections [9, 21, 26, 43] on suicidal patients in a psychotherapeutic context assume that transference of suicidal patients towards clinicians is imbued with hate, projected on the clinician, who then reacts with negative countertransference reactions such as anger, helplessness, guilt, and anxiety. If not recognized and controlled, these reactions can lead to a distorted relationship with the patient [26], and inappropriate and potentially harmful attitudes such as transgression of professional boundaries, rejection, exercise of unnecessary control [9, 43], or premature discharge [33]. A systematic review of quantitative studies [22, 23] concluded that mental healthcare professionals’ countertransference towards suicidal patients mainly involves reactions such as disinterest, anxiety, confusion, helplessness, rejection, inadequacy or entrapment, and less often engagement and fulfillment.

Cognitive-behavioral theorists have also sought to explain therapists’ emotional reactions to suicidal patients, often in ways that align with earlier psychodynamic perspectives [4]. Rudd and Joiner [34] suggest that dysfunctional beliefs held by therapists in the face of suicidality can trigger automatic negative thoughts and maladaptive behaviors such as excessive involvement or premature disengagement from treatment. Cureton and Clemens [3] integrate psychodynamic and cognitive-behavioral frameworks to argue that therapists may suppress less socially acceptable emotions—such as anger or guilt—while expressing only more acceptable ones like sadness. This emotional repression, they contend, can negatively impact the therapeutic alliance.

Qualitative studies [1, 10, 13, 13, 14, 14, 27, 31, 38] showed that mental health professionals caring for suicidal patients experience uncertainty, anxiety, difficulty to establish a relationship and assume responsibility for the patient’s life, as well as helplessness and fear of consequences.

Michaud et al. [24] concluded that the presence of suicidal ideation elicits significant countertransference reactions from the therapist after a single encounter, and that these reactions differ from those generated by a patient with a personality disorder. Nevertheless, only a few studies specifically focus on how the clinician’s reaction varies depending on the suicidal patient’s characteristics. Smith et al. [36] propose a theoretical model of clinicians’ reactions to suicidal patients, which postulates that certain characteristics of the patients provoke three distinct "dysregulated" responses: ambiguity about the sick role (are patients really sick or just seek attention?), need to respond associated with uncertainty about what to do, and fear that, independent of actions taken, outcomes will potentially be fatal. However, there are no studies that have delved into how the clinician’s reactions are rooted in the therapeutic relationship. We therefore aimed to explore not only clinicians’ reactions but also the way these reactions are influenced by specific characteristics of the clinician and the patient.

## Methods

### Data collection

Eighteen healthcare professionals were interviewed by the last author as part of a research project exploring their experiences with individuals in suicidal crisis. Interviews involved physicians, nurses, and social workers, working in the inpatient unit and outpatient center of the Douglas Mental Health University Institute in Montreal, who have regular contacts with suicidal patients. All participants in the study were informed about the research objectives and procedures by the interviewer. Consent forms were distributed, signed, and collected prior to participation, ensuring that all individuals fully understood their involvement in the study.

Since narratives allows for a more straightforward depiction of the complex interplay between emotions, cognitions, volitions, and actions [39], the interviews were inspired by the free association narrative interview model [18]. Respondents were asked to recount two encounters with a suicidal patient, one related to their most recent experience and one that had left a lasting impression. These encounters were explored in-depth through open-ended questions (Table 1). The interviews, lasting 60 to 90 min, were audio-recorded and transcribed verbatim.

**Table 1:** Examples of frequent follow-up questions during interviews

At that moment, what emotions did you feel? What did you think? What did you say? What did you do? How do you assess the quality of your work during that encounter? If you had to do it over, what would you do differently? Did you consider the possible impact on you if the patient were to die by suicide? Why did you choose to discuss this patient? In the encounter you shared, are there any aspects of your own history that influenced the way you reacted?	

We hypothesized that variations in training and professional roles can significantly shape healthcare providers’ perspectives on suicidal patients. Physicians, in particular, often hold primary responsibility for clinical decision-making, placing them in a unique position of accountability that can amplify emotional responses and ethical dilemmas. Their training within a biomedical framework could further influence how they perceive suicidality and approach the therapeutic relationship. To minimize variability and achieve a more focused, nuanced analysis, we chose to concentrate exclusively on physicians in this study.

The respondents included five men and one woman, with varying levels of professional experience. Some were still in specialty training or had recently completed it, while others had several years of clinical practice. They worked either in inpatient psychiatric units or in outpatient services, and all participated in psychiatric emergency shifts. As a result, they had regular contact with suicidal patients both during their routine clinical work and while on duty in the psychiatric emergency service.

### Qualitative data analysis

To analyze the material, we relied on the theoretical model of Hayes [15]. Originally designed for psychotherapy, Hayes’ structural theory of countertransference emphasizes five factors: origins, triggers, manifestations, effects, and management factors. Origins refer to therapists’ areas of intrapsychic conflict. Triggers are therapy events that touch on or elicit the therapist’s origins, e.g. patient’s presenting problems or presentation styles. Manifestations are therapist behaviors, thoughts, or feelings that result from the triggers. Effects are the ways in which countertransference manifestations promote or hinder therapy process. Management factors are therapist behaviors and characteristics that help therapists regulate and productively use their countertransference reactions.

In Hayes’ model, countertransference is defined as any reaction of the clinician to the patient -whether conscious or unconscious, whether in response to the patient’s transference or to other factors. This totalistic definition has been proposed as a framework for understanding clinicians’ emotional responses to patients, both in psychotherapy [16] and in broader clinical contexts [28].

We analyzed the data in two steps. In the first step, we sought to identify sections of the material corresponding to one of Hayes’ five factors. We coded as origins of emotional reactions everything the physician said when discussing their relation to their profession, expectations, fears, perceptions of their role in the healthcare system, perceptions of suicide, as well as their life history and past experiences with suicidal patients. In accordance with the notion of intrapsychic conflict, we tried to identify needs, values, or perceptions that were mutually in conflict, that is, opposed or incompatible. We coded as potential triggers everything the physicians mentioned when describing interactions with patients as well as the context of these interactions, such as the patient’s problems and attitudes, suicidality, and interactions with other healthcare professionals and the patient’s family. We coded all reported physicians’ behaviors, thoughts, and emotions during their interactions with patients as manifestations of emotional reactions. We did not find material sections corresponding to effects, and we found very few excerpts corresponding to management factors, which is why we did not retain these two groups in the analysis. We thus had three groups of material: origins, triggers, and manifestations. Thereafter, we conducted a thematic analysis of the material for each group, which we further separated into units of meaning, which were assembled into subgroups.

In the second step, we examined the relations between origins, triggers, and manifestations. As mentioned, Hayes’ model assumes that a countertransference manifestation emerges when a trigger touches a therapist’s origin. We thus sought to identify patterns linking triggers, origins and manifestations. Since some participants discussed more than two encounters (referring, for example, to a past encounter that influenced the present one), while others did not mention manifestations, or addressed multiple manifestations corresponding at times to different points in the patient’s care, we included all reported encounters that contained at least one manifestation (n = 15). As manifestations are, according to Hayes structural theory, the most superficial and perceptible expression of countertransference, we began by analyzing them and sought for each one a pattern linking it to its origins and triggers.

The first step was conducted by the first and last authors, who analyzed the material separately and discussed disagreements until a consensus was reached. The second step was conducted jointly by the first, second and last author.

## Results

### Origins, triggers and manifestations

This section of the results presents the first step of the qualitative data analysis focused on origins, triggers and manifestations. Given that the manifestations are the most visible part of the emotional reactions, we will begin presenting the results with the manifestations and then move on to the origins and triggers. To illustrate these results, we will include selected excerpts from the interviews conducted with the participants. Each participant has been assigned a code name: Noemie, Hadrien, Paul, Marc, Lionel, and David.

#### Manifestations

We identified three physicians’ manifestations that encompass their thoughts, emotions, and behaviors towards patients. The three manifestations had a dual polarity resulting in six types of reactions.

##### Emotional connection/avoidance

Emotional connection was identified when physicians recognized patient’s emotions and suffering. In these situations, physicians expressed sympathy, sadness, and, in case of positive outcome, relief.

*I felt sorry for him, I thought, "Why does all this happen to such a good person?" The sessions were very heavy. But at the same time, I hoped that talking with him might help. (Noemie).*

On the other hand, physicians sometimes experienced negative emotions, primarily fear and anger, mostly in situations where they felt threatened, manipulated, or unappreciated.

*I was angry because for me, what he was doing was an abuse of the system and of our efforts. We were trying to help him while he himself was doing nothing to move forward. (Hadrien).*

In these situations, they did not invest in the therapeutic relationship and carried out their work without genuine engagement, what we called emotional avoidance.

Sometimes we have such negative feelings towards the patient that we don’t even think, "I could do this or that to help". I think that was the case with this patient. (Hadrien)

##### Confidence/Doubts

The second manifestation pertained to the physician’s level of confidence in their decision-making ability. With some patients, physicians could decide on the best actions to take based on the available information and had confidence in their decision.

It was a really easy decision. I could justify it both clinically and ethically. (Noemie)

In contrast, physicians might consider the available information to be contradictory or ambiguous, and they struggled to decide. They experienced uncertainty and doubts.

*She had been talking about suicide for a long time, and recently, suicidal thoughts became more intrusive. But at the same time, there was no imminent risk. So, how can I decide when to intervene? (Paul).*

It was often at this moment that challenging choices regarding involuntary hospitalization versus alternative treatments should be made, with coercion potentially undermining therapeutic alliance.

*I didn’t want to intervene too aggressively for fear of breaking the relationship with the patient. But at the same time, I was afraid that if she would die by suicide, someone would review the file and say, "Why didn’t you do anything? She was talking about suicide." (Paul).*

##### Powerlessness attributed to own limitations/to the patient

The third manifestation concerned physicians’ perception of their interventions, while feeling stuck in the treatment process. The binary aspect here involved the way physicians explained the impasse. First, the impasse was attributed to the physician’s feeling of not having enough means to help the patient.

*I think that when he was discharged, the suicide risk remained very high. But there was nothing more we could do. […] He wasnt particularly depressed, he was going back to the same harmful environment. He had borderline personality traits, so he could have benefited from psychotherapy, but there was no psychotherapist in his village. (Hadrien).*

In this case, physicians expressed a sense of failure, powerlessness, and discouragement.

*There was no improvement; we kept him in the hospital just so that he wouldnt jump in front of the subway, and we pumped him with medications. So, the most intense feeling I had was actually powerlessness. (Noemie).*

Second, the impasse resulted from patients’ attitudes, and physicians attributed them a part of the responsibility.

*I wanted to show him that there was a gap between what he said and what we saw. So, I told him, "You’ve been in the hospital for a while now, and there’s been no progress. So, I think you’re simply making threats to get something. But that’s not the best way to get what you want". (Hadrien).*

#### Origins

We identified three clinicians’ needs orienting their relationship with suicidal patients. Associated specific challenges often hindered their fulfillment.

##### Need to help

The first of the physicians’ needs was the need to help by developing a relationship where they could provide patients with resources and emotional support.

Most of the time, my initial reaction is to help, to give hope. I want to say something like, "Don’t worry, we will help you get through this.” (Marc)

Nevertheless, with suicidal patients, there were two situations where physicians did not feel they were in a genuine helping relationship. The first was when patients refused help, or when physicians themselves believed that the patient’s request for help was not sincere but rather a way to exploit them.

*There are some patients who, even if they dont really need hospital treatment, do everything possible to get hospitalized. And the surest way to get hospitalized in psychiatry is through suicidal ideation. (Hadrien).*

The second situation was when physicians perceived their patients’ suicidal thoughts as disproportionate to the suffering they observed.

*I have never had a patient for whom I thought, "He is right, given his situation, the only solution left is death." Maybe it would be different if I worked with elderly people or patients with incurable illnesses. (Lionel).*

##### Need for security

Clinicians were aware that their patients’ lives were at stake, which heightened their concern about the potential harmful effects of making a wrong decision—not only on their patients but also on their own career and reputation. Consequently, they wanted to act in a way that avoided any risk for both, themselves, and their patient.

*The psychiatrist’s main interest is to avoid problems. If we let someone go and they die by suicide, everyone will say, "Why didn’t you make a better risk assessment?". (Hadrien)*

However, they understood that zero risk did not exist for reasons related to the suicidal patient, as well as to the nature of the evaluation. First, the information that patients share was not always perceived as credible.

*The most challenging part of the job is assessing suicide risk when a patient is not authentic. It is impossible to be certain about assessment’s validity. (David)*

Second, there were no objective tools to assess suicidality, and therefore, the evaluation often remained a matter of judgment.

*I have a responsibility to evaluate something that is not easy to evaluate. We cannot stage suicide risk the same way we would with cancer. (Hadrien)*

##### Need for efficacy

Finally, physicians wanted to be efficacious, meaning they aimed to achieve specific outcomes in their work with suicidal patients. However, they often experienced a sense of inefficacy.

*We begin residency with a lot of hope to help people, but we quickly come up against the system’s limits. So, the biggest challenge is accepting the limits. (David)*

Their sense of inefficacy was tied to two beliefs about suicidality. The first was that modern psychiatry tends to pathologize all forms of suicidality, often compelling physicians to care for patients for whom treatment offers no real benefit.

*In the past, there was a more medical aspect to psychiatry. But with each new DSM version, there is a kind of expansionism. We have medicalized things like personality disorders, binge-eating… And I feel that the same thing has happened with suicide. (Hadrien).*

Second, they believed that if a patient truly wishes to die, there is little they can do to prevent it.

*Nowadays, there is a perpetual struggle against suicide. I think we should not have this responsibility as psychiatrists. There are hopeless cases and we are not Gods. At the end of the day, if someone wants to commit suicide, they will do it. (Noemie).*

In both cases, they felt that the healthcare system assigns them responsibility without providing them with the means to fulfill it.

#### Triggers

We identified five triggers of emotional reactions: 1) patient’s adverse life experiences, 2) symptoms, 3) suicide risk, 4) patient’s engagement and motivation, and 5) influence of a third-party.

##### Adverse life experiences

Physicians’ understanding of patients’ life conditions was dominated by a perception of present or past adversity, which might involve traumatic events, social precarity, or physical illness.

*He did not have an easy life. He lived in shelters and foster homes. (Paul)*

The perception of patients’ relationships with significant persons was often shaped by a lack of emotional support, neglect or abuse by loved ones.

*He felt really abandoned by his family. I asked if his parents came by, and he replied that it was the first time in his life that his father hugged him. (Lionel)*

##### Symptoms

Interviewed physicians tended to have a binary perception of medical conditions. On the one hand, there were patients with clear-cut depressive, manic, or psychotic symptoms.

*He was very depressed. I had no doubt. In fact, I have rarely seen a depression like this. (Noemie)*

On the other hand, there were patients without symptoms of acute psychiatric illness but with pathological personality traits.

*He didn’t appear depressed. He seemed normal, but he had some personality traits that were not visible at first glance. A lot of passivity, dependence… (David)*

##### Suicide risk

Degree of suicidality and its evolution captured physicians’ attention: when patients had a concrete plan and appeared determined, risk was considered high.

*He had very intrusive ideas of hanging himself. So, he went to the basement, tied a rope to a beam, stood on a chair… Eventually, he got scared and didn’t do it. But he was a patient who would most likely kill himself if we let him go. (Noemie).*

In other situations, when patients had chronic suicidal ideation or when physicians found their statements insincere, risk was considered low.

*He was a young man who was spending his time in the unit playing cards, telling jokes, who got involved in a relationship with another patient. For him, being in the hospital was like being on vacation. But every time I met him, he would say, "I’m desperate, I only think about suicide". (Hadrien).*

Physicians sometimes perceived a potential for self-harm, but assessing immediate risk was difficult due to patients’ ambiguous statements or contradictions between their current and past state.

*I had two contradictory sources of information. When I read the letter, I thought, "This is an involuntary hospitalization, no negotiation". However, I had someone in front of me who said he no longer had the same thoughts as the day before. (Marc).*

However, physicians also reported situations where, regardless of the degree of initial risk assessment and despite treatment, suicidal ideation persisted, or patients engaged in self-harm.

*He attempted suicide after two months in the hospital. So, we went back to square one. How can we consider hospital discharge under these conditions? (David)*

##### Patient’s engagement and motivation

Patients’ overall way of engaging in the relationship with their physician and in the treatment process was among the triggers. Relationally, physicians sometimes perceived patients as genuinely connecting with them and sincerely sharing their experiences and feelings…

*I was struck by the shame, the simplicity… It was inconceivable for him to have reached that point (attempting suicide). (Noemie)*

...and, regarding the treatment, in some cases, patient and physician aligned on the same treatment approach.

*She had experienced several depressive episodes, so she knew well her illness. It was an easy decision for her. When I talked to her about hospital admission, she said, "Okay, this is it, I’m ready to go to the hospital." (Noemie).*

In contrast, some patients hindered physicians from establishing a relationship, often by displaying indifference and insulting them

*He was arrogant, aggressive, cynical. I thought that if I were in his place, if I were 18 years old and facing an older doctor, I would be very respectful. (Lionel)*

… or by making treatment requests that conflict with the physician’s judgment—most commonly regarding hospital admission. These involved patients insisting on being admitted when physicians saw no justification for it or refusing hospital care recommended by the physician.

*I felt some pressure, as if he was trying to convince me that he wasn’t suicidal so that I would let him go. Every time I tried to address other topics, he would say, "I’m not suicidal." (David).*

##### Influence of a third-party

Finally, physicians’ understanding of their patient’s situation was sometimes shaped by input from other healthcare professionals or the patient’s friends or family, whose perspectives might either reinforce or challenge the physician’s judgment.

*I spoke with the social worker, and I told her that I felt comfortable letting the patient go. However, she told me that she was a bit worried. So, I tore up the discharge prescription I had already prepared and said to her, "OK, I will go reevaluate him. If you’re not comfortable letting him go, I’m not comfortable either." (Paul).*

Origins, triggers, and manifestations are summarized in Table 2.

**Table 2:** Groups of origins, triggers and manifestations

Origins	Need to help Need for security Need for efficacy
Triggers	Adverse life experiences Symptoms Suicide risk Patients engagement and motivation Influence of a third party
Manifestations	Emotional connection/avoidance Confidence/doubts Powerlessness attributed to own limitations/to the patient

### Relations between origins, triggers, and manifestations

This section presents the second step of the qualitative data analysis, in which we searched for specific patterns linking origins, triggers, and manifestations in all encounters (N = 15) described by the six participants.

Each manifestation was specifically linked to one of the origins, either explicitly by the physician or through semantic proximity in the physician’s discourse: "emotional connection/avoidance" was linked to the "need to help"; "confidence/doubts" was linked to the "need for security"; " powerlessness attributed to own limitations/to the patient" was linked to the "need for efficacy."

Furthermore, each of the six types of reactions resulting from the dual polarity of the three manifestations was associated with one or two trigger panels, forming seven distinct patterns.

**Fig. 1:**
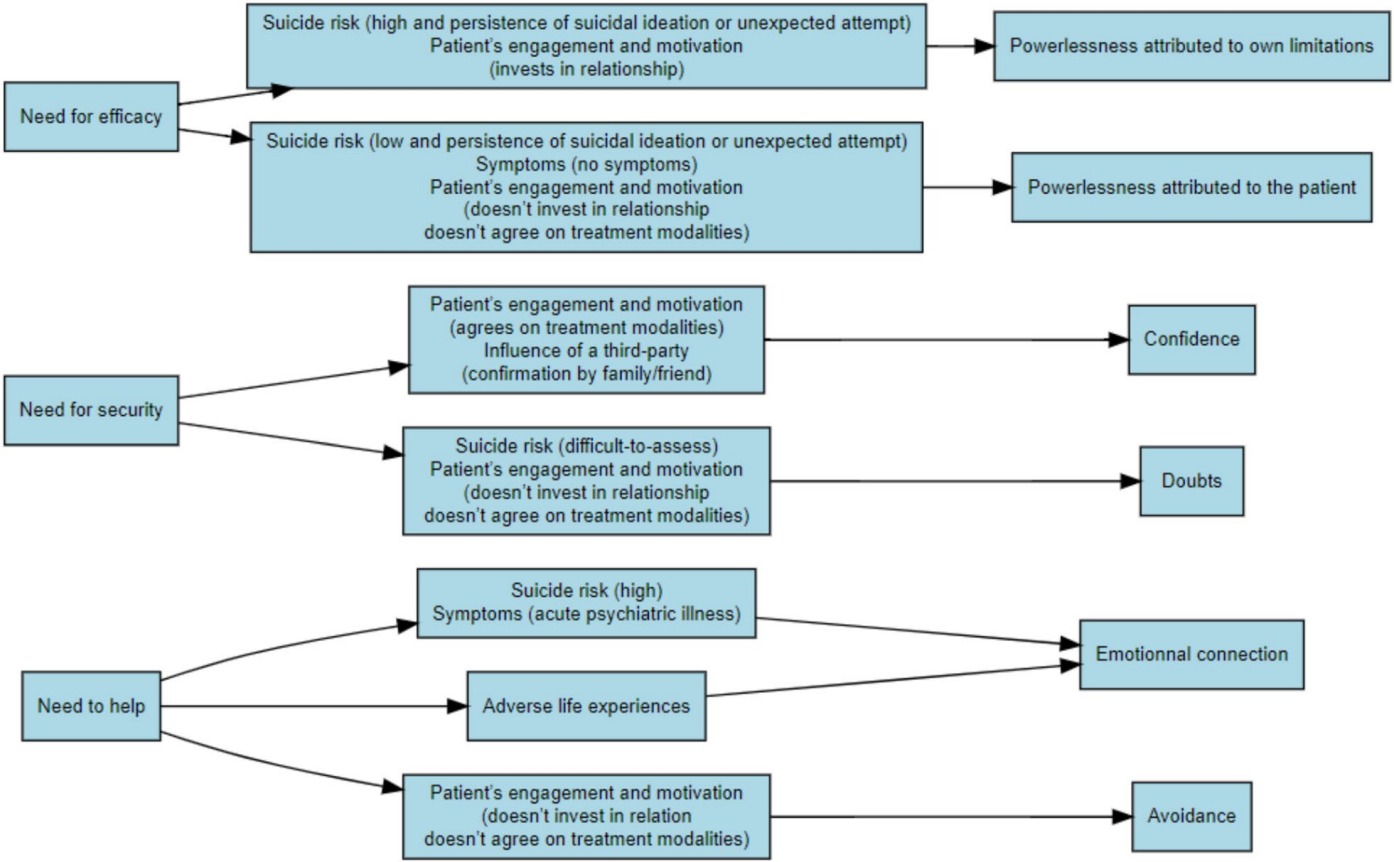
Flowchart showing how specific origins (left) interact with defined trigger panels (center) to generate distinct manifestations (right)

Figure 1 summarizes the 7 patterns while the set of patterns for each encounter grouped by manifestation are summarized in the Additional file 1.

Summaries of an encounter for each of the seven patterns of origins-triggers-manifestations illustrate associations of the three components within each triad. Since the manifestations result from a combination of origins and triggers, we represent this with the formula Origin X + Trigger(s) Y = Manifestation Z.

**Need to Help + (High Suicide Risk + Symptoms of Acute Psychiatric Illness) = Emotional Connection**

Noemie was a young physician deeply committed to making a difference in her patients’ lives. However, she often struggled to understand patients dealing with suicidal thoughts. During a consultation at a general hospital, she met a man suffering from severe depression, who revealed that he had recently attempted suicide by hanging, abandoning the attempt at the very last moment. Noemie was struck by the intensity of his symptoms and convinced that the patient was at a high risk of suicide. She felt the patient’s pain and despair, a reaction that sharply contrasted with her usual inability in connecting with suicidal patients and felt determined to protect him.

**Need to Help + Adverse Life Experiences = Emotional Connection**

Providing emotional support to patients was a core component of Lionel’s professional identity. Nevertheless, he believed that no amount of grief, no matter how great, could justify suicide, and had therefore a difficult time empathizing with suicidal patients. During one of his shifts, Lionel evaluated a young man who had been taken to the hospital by a friend after attempting to jump into a river. Lionel initially experienced a negative emotional response to the patients arrogant and hostile demeanor. The patient talked candidly, during the interview, about his strained relationship with his family and his intense sense that his parents did not care about him. That enabled Lionel to interpret the patient’s suicide attempt as a desperate attempt to communicate his suffering to his parents. Reflecting on his own relationship with his father, Lionel felt a surge of empathy for the patient and a strong desire to support him through his suffering.

**Need to Help + Low Patients Engagement/Motivation = Emotional Avoidance**

Hadrien firmly believed that, even when they dont truly need treatment, some patients can go to great lengths to get admitted to a psychiatric hospital. According to him, the "safest and easiest" way for these patients to get admitted was to express suicidal thoughts. He therefore took care of this group of patients out of obligation and feeling that his efforts to help them were futile. He followed a patient at a psychiatric hospital who appeared to have no significant symptoms and seemed relaxed and at ease on the unit. However, in their one-on-one sessions, the patient portrayed himself as highly depressed and suicidal. The patient resisted Hadrien’s suggestion to discharge him, implying that if he left the hospital and later took his own life, it would be Hadrien’s fault. Hadrien interpreted the patients behavior as an attempt to manipulate him and this perception left him feeling angry.

**Need for Security + (High Patients Engagement/Motivation + Confirmation by Family/Friend) = Confidence**

For Marc, suicidal patients often have conflicting feelings about treatment. He knew that, if he had any doubts about a patients sincere commitment to treatment, his duty was to put the patients safety first. At a community center where he worked, Marc encountered a patient who had written a suicide note to his family the day before. During the interview, the patient said very little, but denied any suicidal thoughts and expressed a willingness to begin outpatient therapy if necessary. Marc made the decision not to hospitalize him but he was not totally convinced of the patients sincerity and, afterward, he felt doubts whether he had made the right choice. The next morning, the patient returned to the community center as planned, accompanied by a friend. Marc observed that the patient had followed the crisis plan, and the patients friend confirmed that everything had gone well. These signs reassured Marc and restored his confidence in the patients commitment and in his own decision to provide outpatient care.

**Need for Security + (Suicide Risk Difficult-to-Assess + Low Patients Engagement/Motivation) = Doubts**

David, a young physician, struggled to assess a patients suicide risk when there were doubts about the patients authenticity. He encountered a man who had arrived to the psychiatric emergency department after having texted his supervisor about having suicidal thoughts. However, during the interview, the patient denied any such thoughts stating repeatedly that he was fine and only wanted to return home. Already conflicted due to the contradiction between the text message and the patients statements during the interview, David felt that his patient was not cooperating authentically, leading to significant uncertainty about how to proceed.

Need for Efficacy + (High Suicide Risk + Persistence of Suicidal Ideation or Unexpected Attempt + High Patients Engagement/Motivation) = Powerlessness Attributed to Own Limitations

Noemie often felt that society imposes a relentless battle against suicide, and that, if a patient is truly determined to die, there is not much that can be done. She followed an adolescent at the hospital, who had heavily invested in his relationship with her. Despite trying various treatments, the patient remained highly symptomatic and suicidal, and shortly after his discharge from the hospital, he attempted suicide. Throughout his hospitalization, Noemie had struggled with a profound sense of helplessness, and when she learned of the attempt, she was overwhelmed by the belief that there was nothing she could have done to help her patient get better.

**Need for efficacy + (Low Suicide Risk + Persistence of Suicidal Ideation or Unexpected Attempt + Absence of Symptoms + Low patient’s engagement/motivation) = Powerlessness Attributed to the Patient**

Paul felt that the healthcare system, driven by the need to avoid legal consequences, often places on physicians the responsibility for managing suicidal patients without psychiatric disorders. He was convinced that these cases fall outside his purview. A suicidal patient he followed at the hospital had a personality disorder and chronic, though low, suicide risk. The patient often rejected proposed therapeutic options and, when upset, would imply that leaving the hospital without a satisfactory solution to his social problems might lead to his suicide. Paul was frustrated by the patients behavior that he perceived as immature and eventually told him that the challenges in his life were his own responsibility to address.

## Discussion

The primary aim of this study was to understand how physicians reactions towards suicidal patients were linked to their own characteristics and to those of the patient. Interviews with physicians were analyzed using Hayes structural model of countertransference. Through this analysis, we identified key elements encompassing manifestations, origins, and triggers emotional reactions. Specifically, we identified three manifestations (emotional connection/avoidance, confidence/doubts, powerlessness attributed to own limitations/to the patient), three origins (need to help, need for security, need for efficacy) and five triggers (adverse life experiences, symptoms, suicide risk, patients engagement and motivation, influence of a third-party). Subsequently, we identified seven patterns of reactions linking triggers on the patients side with origins and manifestations on the physicians side.

The manifestation of "Emotional Connection—Avoidance" was associated with the physicians need to help. Physicians showed opposite tendencies in their emotional engagement with patients, oscillating between forming deep emotional connections and avoiding emotional investment.

High suicide risk in a patient with acute psychiatric symptoms as well as adverse life experiences triggered emotional connection. These triggers may help mitigate the two primary challenges to the physicians desire to help.: (1) the perception that some patients are not genuinely seeking help, and (2) the belief that certain suicidal thoughts are disproportionate. Indeed, symptoms and life adversity may enable physicians to intellectually understand the patients suicidality without becoming emotionally overwhelmed by their patients lives [31] and without getting entangled in often difficult human interactional aspects of the situation [17]. Moreover, perceiving patients as having less control over their personal circumstances or illness prevent physicians from adverse judgement [2].

Conversely, emotional avoidance was linked to the patients disagreement with the physician, or to the patients lack of investment in the therapeutic relationship. Ambivalent or openly hostile patients can challenge the physicians ability to maintain a connection. In such cases, building a closer relationship may help physicians reframe the patients behavior as "acting out" [38]. However, when patients fail to engage or reciprocate efforts to build rapport, clinicians may feel discouraged [41]. This can lead to a vicious cycle of mutual rejection [38], fostering negative emotions and emotional avoidance. To summarize, physicians are more likely to connect with suicidal patients when they can make sense of the patients behavior and perceive the patient as a “good” patient—one who appears grateful, motivated, and aligned with their expectations.

The manifestation of "Confidence-Doubts" reflected physicians underlying security needs. When working with suicidal patients, physicians were particularly concerned about potential consequences stemming from their decisions. This insecurity was related to their difficulty in fully trusting patients and to the lack of effective assessment tools. For instance, physicians are often haunted by the prospect of a suicidal patient with a "hidden agenda" who knows what they need to say in order to grant hospital discharge [42]. Compounding this challenge is the finding that some clinicians perceive suicide risk assessment as an inexact science [27] and consider traditional rating scales to be of limited relevance for informing clinical decision-making [42], a view that is consistent with existing guidelines [29] and research findings [6].

In light of these limitations, physicians reported relying on their intuition when making assessments, even while acknowledging the possibility of error. This reliance on intuition aligns with findings from previous research [13, 14]. Notably, although the study did not specifically explore the use of structured tools, collaborative approaches such as the Collaborative Assessment and Management of Suicidality (CAMS [20],) may have offered a valuable alternative. By fostering clinician–patient dialogue and reducing clinician stress, such frameworks could have supported respondents in navigating the uncertainty they described in their clinical decision-making.

As with challenges related to the need to help, specific triggers— patient agreeing with treatment modalities and receiving confirmation from a third party—promoted confidence over doubts. Hagen et al. emphasize the importance of the working relationship, in other words a results-oriented relationship, in dealing with suicidal patients, [13, 14]. A strong working alliance thus seems to mitigate the challenges that undermine physicians sense of security. In addition, as in line with our results, previous research showed that talking to colleagues and to persons close to the patient are common strategies for reducing uncertainty [42].

By incorporating both the patients perspective and input from third parties into the decision-making process, physicians share control within the therapeutic relationship. This collaborative approach stands in stark contrast to situations involving patients who fail to engage in the therapeutic relationship, disagree with treatment modalities, or present an immediate suicide risk that is challenging to assess. In such circumstances, physicians may struggle to maintain confidence, further complicating their ability to navigate the therapeutic process effectively.

In such situations, physicians often feel unable to rely on the patients cooperation, leaving the burden of decision-making solely on their shoulders. This responsibility can lead to significant doubts and difficult dilemmas, such as deciding whether to pursue involuntary hospitalization—a course of action that may cause patients to perceive the clinician as infringing on their autonomy. Exerting control over a patient, while sometimes necessary, can jeopardize the therapeutic relationship and leave clinicians with a sense of discomfort. Gilje et al. [10] describe this as the paradoxical nature of control when working with suicidal patients: having control does not necessarily provide clinicians with a true sense of safety. In summary, confidence and control emerge as fundamental driving forces when physicians confront the inherent uncertainty of managing suicidal crises.

The manifestation of " Powerlessness attributed to own limitations/to the patient " was associated with physicians need for efficacy in their work. Physicians faced two main challenges in this regard: the perception of a healthcare system that tends to overly pathologize suicidality and their own sense of helplessness when treating patients determined to end their lives, based on the belief that such decisions are ultimately irreversible.

Suokas and Lönnqvist [37] propose that clinicians wish to be powerful could be a way to avoid enduring the empathic pain of suicidal patients hopelessness. Consistent with this view, our findings revealed that physicians often oscillate between two conflicting tendencies: a willingness to take responsibility for saving the patients life and a drive to consider the patient capable of taking responsibility for their own decisions. This dynamic has been previously reported in the literature [17]. Our study further highlights the significant role of patient characteristics as triggers that influence this dual movement.

Both the powerlessness physicians attributed to their own limitations and the powerlessness they attributed to the patients were linked to cases where suicidal thoughts persisted despite treatment. When physicians encountered patients with low suicide risk, no acute psychiatric symptoms, and minimal engagement in the therapeutic process, they often questioned the appropriateness of treating such individuals within a psychiatric framework. This skepticism stemmed from the systems perceived overpathologization of suicide. In these instances, physicians tended to view the patients as immature or irresponsible, attributing the lack of treatment progress to the patients themselves.

Previous research supports this pattern. Clinicians have been shown to question the authenticity of some patients suicidality, particularly in cases where suicidal ideation is expressed without progressing to life-threatening self-harm [1] or where patients do not “adamantly assert” their intent to harm themselves [19]. Such patients are often perceived as illegitimate users of healthcare services and, at times, manipulative. Additionally, when suicide risk is assessed as low and no significant psychiatric symptoms are present, physicians may believe the patient is in control of their suicidal thoughts and overall situation.

As Redley [31] suggests, physicians who view suicidality as a willful act sometimes make moral judgments about their patients, even confronting them. This reveals a shift from medical-psychiatric frameworks of understanding to moral categorizations. Conversely, when physicians assess a high suicide risk, they are more likely to interpret suicidal thoughts as symptoms beyond the patients control, requiring immediate intervention. Moreover, the patients genuine emotional investment in the therapeutic relationship can dispel concerns about manipulation. Again, manipulation is not seen as a pathological characteristic but rather as a perceived moral failing.

However, even when patients demonstrate commitment to the therapeutic relationship, the persistence of suicidality despite treatment efforts can leave physicians feeling powerless. This sense of impotence may reinforce the belief that for individuals resolutely intent on dying by suicide, interventions have limited efficacy. Clinicians who feel personally responsible for managing suicidality may experience therapeutic failure as a direct threat to their professional identity [38]. This can lead to fatalistic or defeatist attitudes, viewing suicide as “inevitable and untreatable” [1]. Such a stance effectively neutralizes the problem by attributing it to destiny.

In this study, clinicians convictions about certain characteristics of suicidal patients, their own role in caring for them, as well as views on risk assessment and the healthcare system, interact with patient-specific and contextual factors during evaluations to give rise to particular emotional reactions. Within the framework used for our analysis—which adopts a totalistic definition of the psychoanalytic concept of countertransference—such assumptions can be understood as the cognitive expression of deeper intrapsychic conflicts. From this perspective, they may be interpreted, following Maltsberger and Buies [21] theoretical model, as unconscious defenses against feelings of impotence and fear in the face of suicidality. Alternatively, in a cognitive-behavioral framework such as the Therapeutic Belief System (TBS) [34], which has also been applied to understanding clinicians relationships with suicidal patients these assumptions may reflect core beliefs shaped by the clinicians cognitive schemas.

Regardless of the theoretical framework used to interpret these reactions, the main clinical implication of this study is to offer a practical model that helps physicians understand their emotional responses to suicidal patients as co-constructed experiences. This, in turn, can serve as a valuable tool for deepening reflection on the physician–patient relationship and ultimately improving therapeutic engagement.

## Limitations

This study has several limitations. First, while the sample was not intended to be representative and although professionals with varying levels of experience were included, the overrepresentation of males may have influenced the findings by emphasizing certain perspectives over others.

Second, the study sample consisted of physicians who had completed most of their medical training and were practicing in Canada. Their professional culture, perceptions of suicidality, and approaches to psychiatric care were shaped by geographic and cultural factors, as well as by the organization of the Canadian healthcare system and its medicolegal context. As such, the findings may not be fully transferable to other settings, particularly those in countries with significantly different healthcare systems.

Additionally, data analysis and interpretation were influenced by the authors personal experiences and preconceived notions about psychiatric care and suicidality. These perspectives were inevitably shaped by the territorial context (Switzerland) in which the authors practice, potentially introducing bias into the interpretation of the results.

Finally, Hayes structural theory was adopted as a conceptual framework after the interviews had been conducted. As a result, the interview guide was not initially designed with this framework in mind, limiting the studys capacity to fully explore certain aspects of the theory, such as countertransference effects and management factors.

## Conclusions

While there are multiple studies exploring the relational aspects of clinicians interactions with suicidal patients, this study is among the few that focus specifically on physicians. To our knowledge, it is also the first to examine how specific issues related to both physicians and patients shape their interactions. This study provides valuable insights into clinicians emotional responses to suicidal patients and offers a framework that delineates how patient-related issues interact with physician-related issues, ultimately triggering specific emotional reactions. This framework can help physicians understand their reactions to suicidal patients as a co-construction between themselves and the patient. This perspective could enable clinicians to view their emotional responses not as obstacles but as valuable insights into the patients experience.

By deconstructing physicians reactions into their origins, triggers, and manifestations, and analyzing the interplay among these elements, this study offers a straightforward and reproducible methodological model. This model can be utilized in research, clinical supervision, and medical training to facilitate a deeper understanding and reflection on the physician–patient relationship. Building on these findings, future research can further enhance understanding of clinicians reactions to suicidal patients. Such research could contribute to the development of effective strategies aimed at improving care and outcomes for this vulnerable population.

## Supplementary Information

Additional file1

## Data Availability

The data that support the findings of this study are available from the corresponding author upon reasonable request.
