# Supplementary material for: Exploring physicians’ emotional reactions to suicidal patients: the impact of physician- and patient-related issues
Source: Ann Gen Psychiatry. 2025 Oct 24;24:65. doi: 10.1186/s12991-025-00602-9 (PMC12553232; doi:10.1186/s12991-025-00602-9)
Supplement: Supplementary file 1 — Additional file1 [file 12991_2025_602_MOESM1_ESM.docx]

**Additional File 1.**

***This file presents detailed results for each of the 15 encounters, organized by manifestation. Most manifestations were linked to an origin, and when no origin was associated with a specific manifestation, it is denoted by a dash (-). For manifestations with an associated origin, the origin consistently belonged to the same group of origins (need to help, need for security, need for efficacy).***

***Each manifestation was linked to 3 to 5 different triggers. Notably, a single trigger panel emerged as nearly universal across participants for most manifestations. The sole exception was the manifestation Emotional Connection, which involved two distinct trigger panels. Triggers that align with the predominant pattern(s) for a given manifestation are highlighted.***

***Consequently, in most encounters where the same manifestation was observed, the origin belonged to the same group, and a single, predominant trigger panel was identified.***

**EMOTIONAL CONNECTION**

***Noemie- 1st encounter***

Manifestation : Understands suffering; sadness; relief; wants to protect

Origin : She often views suicidal thoughts as excessive and pulls away. She invests in care when she feels having a meaningful role. (need to help)

Triggers : Depressive symptoms; High suicide risk; Doesn’t invest in relationship

***Noemie- 2nd encounter***

Manifestation: Sympathy; wants to protect and provide emotional support

Origin: She often views suicidal thoughts as excessive and pulls away. She invests in care when she feels having a meaningful role. (need to help)

Triggers: Psychotic symptoms; High suicide risk; Invests in relationship

***Hadrien- 1st encounter***

Manifestations: Understands suffering; wants to provide emotional support

Origins : -

Triggers : Adverse life experiences; Pathological personality traits; Suicide risk difficult-to-assess; Doesn’t invest in relationship

***Lionel- 1st encounter***

Manifestation : Understands suffering; sympathy; wants to help get better

Origin: He sometimes perceives suicidal thoughts as disproportionate, which hinders his empathy. (need to help)

Triggers: Adverse life experiences*;* Pathological personality traits; Low suicide risk; Invests in relationship

***David- 2nd encounter***

Manifestation: Sympathy; wants to protect and provide emotional support

Origin: When a patient turns to him for guidance, it fosters his understanding and compassion. (need to help)

Triggers: Adverse life experiences*;* Depressive symptoms; High suicide risk; Invests in relationship

**AVOIDANCE**

***Hadrien- 2nd encounter***

Manifestation: Feels threatened and manipulated; fear; anger; lack of engagement

Origin: He struggles to feel like he’s helping when the patient hasn’t asked for help. (need to help)

Triggers: Pathological personality traits; Low suicide risk; Doesn’t agree on treatment modalities; Confirmation by another healthcare professional

***Marc- 2nd encounter***

Manifestation: Feels manipulated; anger

Origin: -

Triggers: Pathological personality traits; Low suicide risk; Doesn’t invest in relationship; Another healthcare professional disagrees

***Lionel- 1st encounter***

Manifestation: Feels unappreciated; lack of engagement

Origin: He sometimes perceives suicidal thoughts as disproportionate, which hinders his empathy. (need to help)

Triggers: Adverse life experiences; Pathological personality traits; High self-harming risk; Doesn’t invest in relationship

***David- 1st encounter***

Manifestation: Feels threatened; fear.

Origin: He emotionally withdraws when the patient rejects his advice. (need to help)

Triggers: Manic symptoms; Suicide risk difficult-to-assess; Doesn’t agree on treatment modalities; Confirmation by another healthcare professional.

**CONFIDENCE**

***Marc- 1st encounter***

Manifestation: Confidence in decision and in available information.

Origin: He prioritizes avoiding risks when working with suicidal patients When the patient commitment to treatment is ambivalent, he feels entirely responsible for their security. (need for security)

Triggers: Adverse life experiences; No symptoms; Suicide risk difficult-to-assess; Agrees on treatment modalities; Confirmation by significant other

***Noemie- 3rd encounter***

Manifestation: Confidence in decision

Origin: She finds it difficult to discern the truth without the patient's cooperation. (need for security)

Triggers: Manic symptoms; High suicide risk; Agrees on treatment modalities; Confirmation by another healthcare professional

**DOUBTS**

***Marc- 1st encounter***

Manifestation: Struggles to decide; contradictory information; considering involuntary hospitalization

Origin: When the patient’s commitment to treatment is ambivalent, he feels entirely responsible for their security. (need for security)

Triggers: Adverse life experiences; No symptoms; Suicide risk difficult-to-assess; Doesn’t invest in relationship

***Lionel- 2nd encounter***

Manifestation: Ambiguous information; considering involuntary hospitalization

Origin: He believes that the lack of objective tools to assess suicide risk makes establishing a connection with the patient crucial in decision-making. (need for security)

Triggers: Pathological personality traits; Suicide risk difficult-to-assess; Doesn’t agree on treatment modalities; Confirmation by another healthcare professional

***Lionel- 3rd encounter***

Manifestation: Uncertainty; considering involuntary hospitalization

Origin: -

Triggers: Adverse life experiences; Depressive symptoms; Suicide risk difficult-to-assess; Doesn’t agree on treatment modalities; Confirmation by another healthcare professional

***Paul- 1st encounter***

Manifestation: Struggles to decide

Origin: He needs to base his decisions on concrete evidence. Impossible to make objective assumptions when evaluating suicide risk. (need for security)

Triggers: Adverse life experiences; No symptoms; Low suicide risk; Doesn’t agree on treatment modalities; Another healthcare professional disagrees

***Paul- 2nd encounter***

Manifestation: Anxiety; uncertainty; considering involuntary hospitalization

Origin: He needs to base his decisions on concrete evidence. Impossible to make objective assumptions when evaluating suicide risk. (need for security)

Triggers: Adverse life experiences; Depressive symptoms; Suicide risk difficult-to-assess; Doesn’t agree on treatment modalities

**David- 1st encounter**

Manifestation: Anxiety; uncertainty

Origin: He feels uncertain about risk assessment when the patient does not cooperate sincerely. (need for security)

Triggers: Manic symptoms; Suicide risk difficult to assess; Doesn’t agree on treatment modalities; Confirmation by another healthcare professional

***POWERLESSNESS ATTRIBUTED TO OWN LIMITATIONS***

***Hadrien- 1st encounter***

Manifestation: Feels unable to move forward

Origin: -

Triggers: Adverse life experiences; Pathological personality traits; Self-harming risk difficult-to-assess; Doesn’t invest in relationship

***Noemie- 2nd encounter***

Manifestation: Feeling of not having enough means; sense of failure; discouragement

Origin: She wants to make a difference in the patient's care, but often there is little to do when a patient truly wants to die. (need for efficacy)

Triggers: Psychotic symptoms; High suicide risk and persistence of suicidal ideation; Invests in relationship

***Paul- 3rd encounter***

Manifestation: Feeling of not having enough means; sense of failure

Origin: With suicidal patients, many interventions are ineffective and are performed purely out of obligation. (need for efficacy)

Triggers: No symptoms; High suicide risk and suicide attempt during treatment; Invests in relationship

***David- 2nd encounter***

Manifestation: Feels trapped; discouragement

Origin: -

Triggers: Lack of emotional support; Depressive symptoms; High suicide risk and suicide attempt during treatment; Invests in relationship

**POWERLESSNESS ATTRIBUTED TO THE PATIENT**

***Paul- 1st encounter***

Manifestation: Views patient’s attitude as irresponsible

Origin: With suicidal patients, many interventions are ineffective and are performed purely out of obligation. (need for efficacy)

Triggers: Adverse life experiences; No symptoms; Low suicide risk and persistence of suicidal ideation; Doesn’t agree on treatment modalities; Another healthcare professional disagrees

***Hadrien- 2nd encounter***

Manifestation: Thinks that patient is immature

Origin: The excessive pathologization of suicide makes him feel responsible for patient’s security, yet without the means to effectively handle risk. (need for efficacy)

Triggers: Pathological personality traits; Low self-harming risk and persistence of suicidal ideation; Doesn’t agree on treatment modalities; Confirmation by another healthcare professional
